# Dynamic Intraligamentary Stabilization (DIS) Repair for ACL Ruptures in Pediatric and Adolescent Patients: An Initial Pilot Study with Long-Term Follow Up

**DOI:** 10.3390/children13030393

**Published:** 2026-03-12

**Authors:** Niklaus Schoepke, Tobias Tjalf Krause, Nadine Kaiser, Thorsten Müller, Sandro Kohl, Kai Ziebarth

**Affiliations:** 1 Department of Pediatric Surgery, University Children’s Hospital Bern, 3010 Bern, Switzerland; nik_schoepke@gmx.ch (N.S.); tobiastjalf.krause@insel.ch (T.T.K.); 2 Department of Pediatric Orthopaedics, University Children’s Hospital Bern, 3010 Bern, Switzerland; nadine.kaiser@insel.ch; 3 Interdisciplinary Department of Sport Medicine, University Hospital Bern, 3010 Bern, Switzerland; thorsten.mueller@insel.ch; 4 Traumacenter, Hirslanden Clinic Zuerich, 8008 Zurich, Switzerland

**Keywords:** anterior cruciate ligament rupture, children, adolescents, dynamic intraligamentary stabilization, anterior cruciate ligament repair

## Abstract

**Highlights:**

**What are the main findings?**

- In this pilot series of 22 skeletally immature patients with ACL rupture, dynamic intraligamentary stabilization (DIS) showed potential facilitation of ACL healing with good clinical short term outcomes and satisfactory patient reported outcomes after 11 years.
- There was a high rate of rerupture and patients needing a revision surgery of 55%.

**What are the implications of the main finding?**

- DIS in children carries a substantial risk of failure and should be considered with caution in this population.
- Preservation of the native ACL is conceptually attractive, but controlled comparative studies are required to define its role in skeletally immature patients.

**Abstract:**

**Background/Objectives**: The aim of this study was to report first experiences with dynamic intraligamentary stabilization (DIS) technique for anterior cruciate ligament (ACL) rupture in children and adolescents. **Methods:** A case series of 22 children and adolescents with a mean age of 13.3 years underwent primary repair of an ACL rupture using the DIS technique as an off-label use in skeletally immature patients. Patients were evaluated for laxity, strength, range of motion (ROM), and functional tests, as well as Tegner, Lysholm, International Knee Documentation Committee (IKDC), and PedsQL scores after 3 years. A follow up after 11 years was conducted to analyze long-term results, rerupture rates and reinterventions. **Results**: Three years after surgery, there was no significant difference in laxity, strength, ROM, and in the functional tests comparing the injured to the contralateral knee. The Tegner Index after surgery showed a slight drop of 0.8 points, from 7.1 preoperatively to 6.3. Mean IKDC, Lysholm, and peds-QL scores were 91.17 (range 62.64–98.85, median 94.25), 88.27 (range 58–100, median 93), and 88.78 (range 58.15–100, median 91.30). Overall failure rate of the DIS-repaired knees was 55% (12 of 22 patients). In ten patients, reruptures happened at an average time of 3.2 years after initial surgery; additionally, two patients with chronic instability had to undergo revision ACL reconstruction. **Conclusions**: DIS repair might help ACL healing with satisfactory functional outcomes. However, given the high failure and reintervention rates, further studies need to show non-inferiority of the DIS technique in children and adolescents before being considered a valid treatment option.

## 1. Introduction

The incidence of anterior cruciate ligament (ACL) injury in children and adolescents has steadily increased [1,2,3]. As the recent literature shows, there might be a better outcome after early operative treatment compared to a non-operative approach. The rationale for surgical treatment is restoring stability of the knee and decreasing the risk of secondary knee damage, e.g., meniscal tears or chondral injury [4,5,6,7,8]. This applies for the treatment of ACL rupture in children, too, as shown by Preiss et al. in 2012 [9]. Depending on the age of the patient and maturity of the bone, several surgical approaches are available to replace the ACL in children and adolescents [8,10,11,12]. But a native ACL is not only responsible for mechanical stabilization, it also bears a proprioceptive function. Hence, preservation of a torn ACL after injury would be desirable [13,14]. Steadman reported about healing potential of the ACL in proximal injuries in 13 patients [15]. However, various other attempts to treat ACL injuries by restoration of the ligament failed [10,16]. A study by Schultz from 1998 showed an unstable ACL in 23 of 41 pediatric patients after repair [17].

In 2011, a new technique for the treatment of acute ACL injuries, dynamic intraligamentary stabilization (DIS), was introduced [18]. This technique relies on the self-healing ability of the injured ACL. First experiences of the originators showed promising short-term results in the adult population [18,19]. With this surgical technique, the native ACL was preserved if surgery was conducted immediately after injury. Considering the increased healing potential in children and adolescents compared to adults, the DIS method was introduced in children, as well. In this study, we describe our experience in a single-center series of 22 patients with a long-term follow up over 11 years. The purpose of this study is to analyze the new surgical technique regarding clinical outcome with functional assessments, as well as adverse events such as growth disturbances and revision rate.

## 2. Patients and Methods

After IRB approval, we treated 22 patients between March 2012 and April 2014 at our institution. All patients and their legal guardians were made aware that, for this age group, this was a novel, off-label technique and gave informed consent. All patients presented with an acute unilateral anterior cruciate ligament (ACL) rupture diagnosed by MRI prior to surgery. The cohort consisted of 8 (36%) female and 14 (64%) male children. Mean age was 13.3 years (range 9.33–15.83 years, median 13.92 years) at the time of surgery. All patients had radiologically open physis to some degree at the time of surgery. The injury affected the right side in ten patients (45%) and the left side in twelve (55%) patients. The time from trauma to surgery averaged 15 days (range 2–33 days, median 14 days), and three patients were treated outside of the optimal timeframe of 21 days known for DIS. Only nine patients (41%) had an isolated ACL rupture, while the other 13 patients (59%) had associated meniscal tears (Table 1).

All 22 patients underwent dynamic intraligamentary stabilization (DIS) repair using the Ligamys intraligamentary stabilization screw (Mathys AG, Bettlach, Switzerland). The technique itself was described in detail in the original publication [18].

**Figure 1:**
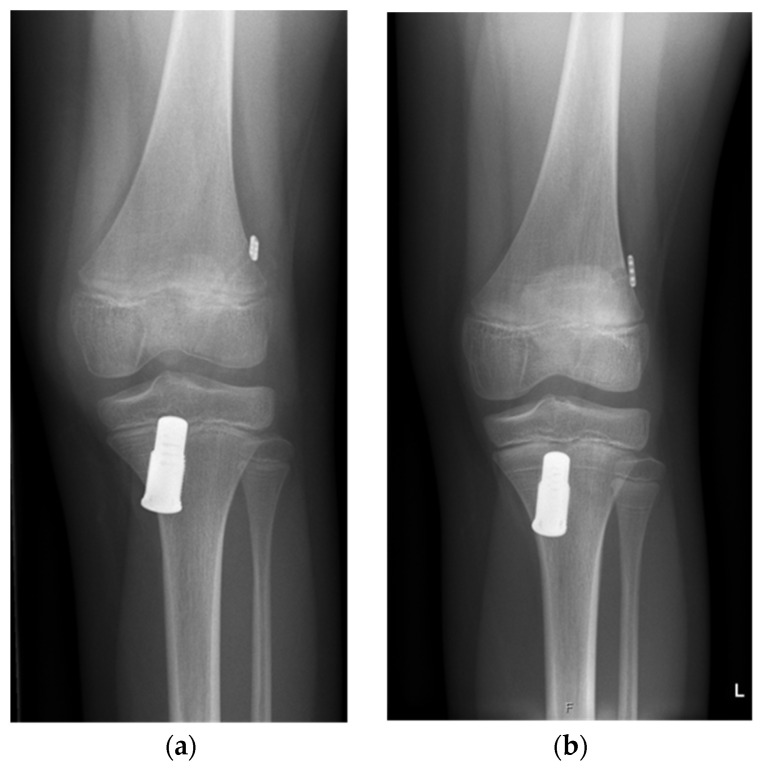
Anterior–posterior radiographs of a knee of an eleven-year-old girl who underwent dynamic intraligamentary stabilization repair. (**a**) Initial postoperative radiograph; (**b**) one year postoperative: Due to the growth of the patient (symmetric Harris growth line), the Ligamys screw is now located more distally. The physis of the proximal tibia is still open.

In a diagnostic arthroscopy, the ACL injury and concomitant lesions were confirmed. In case of a meniscal tear, the meniscus was repaired first, before taking care of the ACL. The torn ACL was prepared with shuttle sutures (2-0 PDS, Polydioxanon) depending on the localization of the rupture: In proximal ruptures, the proximal end of the ACL was prepared with shuttle sutures; in case of intraligamentary lesions, both stumps were sutured. The next step was drilling the respective tibial and femoral tunnels (10 mm and 6 mm) according to conventional ACL reconstruction techniques. On the tibial side, the Ligamys Monobloc holding the spring screw mechanism was inserted. Depending on the age and size of the child, the screw had to be placed through the physis. As mentioned above, in case of intraligamentary lesions, several sutures to the femoral or tibial stump were needed to align the ACL. Finally, a polyethylene braid (1.8 mm) was shuttled from the femoral side through the Ligamys Monobloc on the tibial side. The polyethylene braid was then fixed with an Endobutton, together with the shuttle sutures from the ACL stump on the femoral side. The polyethylene braid was tensioned in the Ligamys screw with a continuous tension of 30–50 N over the entire range of motion of the knee on the tibial side. The polyethylene braid, therefore, prevents anterior tibial translation and distraction of the ACL stumps during the healing phase of the realigned ACL in the first three months postoperatively. Microfracturing of the femoral footprint supports the self-healing process of the ACL. Figure 1 shows the radiograph of an implanted Ligamys system in an 11-year-old girl with screw placement through the physis. All surgeries were performed by the same pediatric orthopedic surgeon (author K.Z.) under the guidance of one of the originators of the technique (author S.K.).

After surgery, all patients followed structured rehabilitation protocols with immediate weight bearing. For the first five days, an extension brace was used for immobilization.

Clinical evaluation followed at 6 weeks, as well as three, six, twelve, and 24 months. Radiological follow up 6 months after surgery included anteroposterior and lateral radiographs to detect possible growth disturbances.

Hardware removal was offered to all patients 6 months postoperatively.

We performed a more thorough follow-up control at an average of 42 months (33–58 months) after surgery in 11 of the 14 patients (79%) without rerupture up to this date. Three of the patients without rerupture did not take part in this follow up: one was from abroad, and two were unwilling to take part in the follow-up control. The follow up included assessments of Tegner, Lysholm, IKDC, and PedsQL scores, as well as measurements of range of motion, muscle strength, and functional tests (single hop test, crossover hop test, 6 m hop test, and triple hop test). Range of motion was measured clinically; laxity was measured using a KT 1000 arthrometer; strength was measured using an isokinetic C (HumacNorm by CSMI). All tests were performed by the same physiotherapist (author T.M.).

**Figure 2:**
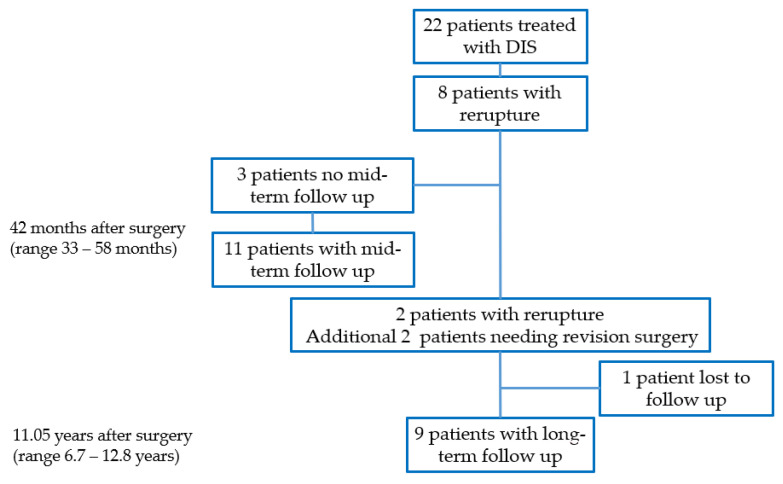
CONSORT flow diagram.

For a long-term follow up, we contacted the remaining 14 patients without a rerupture again. We reached 13 patients, including 2 patients who did not take part in the previous control after 42 months. One patient was lost to follow up. Four of these 13 patients, in the meantime, also needed revision surgery. However, 9 out of 22 patients remained for a long-term follow up after 11.05 years (range 6.67 to 12.83 years, mean 11.33 years). (See Figure 2).

## 3. Results

### 3.1. Short-Term Results

There were no complications in the immediate postoperative course. All patients could follow the structured rehabilitation program as planned. There were no signs of infection, no reported instability, and normal physiological range of motion in the initial follow ups at 3 and 6 months postoperatively. Radiological analysis after 6 months with conventional X-ray, including ap. and lat. radiographs in 21 patients, showed no signs of hardware loosening, growth arrest, or osteoarthritis.

### 3.2. Revision Surgery/Hardware Removal

Overall, we found a failure rate of dynamic intraligamentary stabilization (DIS) repair, defined as the need of a revision/reconstruction surgery, in 12 of 22 patients (55%). Rerupture of the repaired anterior cruciate ligament (ACL) occurred in 10 patients (45.5%), with a mean age of 15.8 years at the time of the rerupture (range 11.9–19.6 years, median 15.9) and a female to male ratio of 2:6. Rerupture happened at an average time of 3.2 years (range 0.9–10.3 years, median 1.9 years) after the initial surgery. All reruptures had a traumatic cause (e.g., knee distortion playing football, on trampoline, skiing, Swiss wrestling, bike accident) after the knee was reported to be stable subjectively and objectively at the routine follow ups after 3 and 6 months. Diagnosis was confirmed by MRI, and all reruptured ACLs were treated with a transphyseal ACL reconstruction using hamstring grafts. One patient with a rerupture 10.3 years after initial DIS had an additional chondral lesion in the MRI, as well as a significant unilateral genu valgum. Therefore, the ACL reconstruction was combined with an osteochondral autograft transfer (OATS) and a varus osteotomy. In two patients, the ACL had to be reconstructed due to clinical signs of instability 4 and 5.25 years after initial surgery. The revision rate was affected by neither delayed DIS repair (DIS repair after 21 days, one of three patients (34%); versus DIS repair within 21 days, 11 of 19 patients (58%)), nor by concomitant meniscal injury (isolated ACL group, five of nine patients (56%); versus ACL plus initial meniscal injury, seven of 13 patients (54%)).

Hardware removal was performed in 18/22 patients. Four patients chose to retain the Monobloc. In 14 patients, elective removal of the DIS screw was scheduled. During Hardware removal, a concurrent diagnostic arthroscopy was performed. Two patients with already-sutured lateral meniscus at index operation needed another refixation of the meniscus on this occasion. In all 14 patients, the ACL was reported to be healed and intact at time of arthroscopy.

Another four patients underwent hardware removal during revision surgery for rerupture.

Two patients (9%) needed a reoperation on the affected knee for persisting problems. One male patient had an accident 15 months postoperative while skiing, at age 15.8 years. The MRI showed an intact ACL, but, due to persistent pain, a knee arthroscopy was performed. Arthroscopic findings showed adhesions between ACL and PCL, which were removed. The second male patient, at age 16.8 years, underwent repair of both menisci after an accident 28 months after initial surgery and 20 months after hardware removal (see Table 2 for detailed patient information).

In total, 29 reinterventions were performed in 18 of the initial 22 patients.

### 3.3. Mid-Term Follow Up

#### 3.3.1. Back to Sports Tests

At mid-term follow up (42 months after the initial surgery, 11 patients without rerupture to this time analyzed), no patients had significant differences in range of motion, laxity, or strength compared to the contralateral side (see Table 3). While flexion and neutral position were symmetric, the difference in extension was only 1.8°, with better extension in the injured knee. Lachmann test showed a slightly larger tibial translation of 1.1 mm in the injured knee. The calculated limb symmetrical index (LSI) for strength was 119.7%, showing the injured knee being stronger at the time of follow up.

Functional tests also showed no significant difference comparing the affected knee to the contralateral side. The results of two tests were slightly in favor of the injured knee (6 m hop and crossover hop tests), and two tests were in favor of the contralateral side (single hop test and triple hop tests) (Table 3).

#### 3.3.2. IKDC, Lysholm, PedsQL and Tegner Score

Subjective clinical scores 42 months after surgery showed overall good results, with the IKDC Score being 91.17 (range 62.64–98.85, median 94.25), and the Lysholm Score 88.27 (range 58–100, median 93). The total peds-QL–score was 88.78 (range 58.15–100, median 91.30), with the psychological subscore being 86.28 (range 62.5–100, median 88.33), and the physical subscore 93.47 (range 50–100, median 100).

The evaluation of activity level using the Tegner index showed a slight drop after surgery of 0.82 (range 0–3, median 0). Tegner before ACL injury was an average of 7.09 (range 4–10, median 7) and, after 42 months, an average of 6.27 (range 2–10, median 7).

### 3.4. Long-Term Follow Up

None of the nine remaining patients without a revision to this follow-up time had any further surgery on the DIS knee.

Two patients (22.2%) found their level of activity reduced compared to before ACL rupture and DIS repair.

Five patients (55%) were completely pain-free, four patients (44%) suffered from infrequent knee pain, and none of the patients had constant knee pain.

Two patients (22.2%) reported a feeling of instability in the affected knee.

None of the patients suffered from a difference in leg length that needed any therapy or intervention.

All but one patient was satisfied with the result of the DIS repair at long-term follow up (Table 4).

## 4. Discussion

We investigated the clinical and functional outcomes of dynamic intraligamentary stabilization (DIS) repair for anterior cruciate ligament (ACL) ruptures in pediatric and adolescent patients. DIS was performed as an off-label application in skeletally immature patients as part of an exploratory pilot series. The present study demonstrates that DIS repair, in some cases, may facilitate ACL healing in skeletally immature patients. All patients maintained subjective and clinical ligament integrity at short-term follow up, 63% of the patients at mid-term follow up, and 36% of the patients after 11 years.

Nevertheless, the overall rerupture rate of 55% in this cohort was substantially higher than reported in adult DIS populations, with 2.8–8% [18,19,20,21,22]. This might be due to a shorter follow-up period in the latter publications. In a recent multicenter study, Senftl et al. showed a rerupture rate of 16.3% after 26 months, and a persistent instability in 18% of adult patients [23], which is still lower than in our cohort. However, overall graft failure and rerupture rate after undergoing any kind of ACL repair or reconstruction is known to be higher in the pediatric population compared to adults. Reported failure rates in children with ACL reconstruction range up to 19% [11,24,25,26], without relevant difference in applied reconstruction method, compared to 4.2–4.4% in the adult population [27,28]. Rilk and Vermeijden showed respective failure rates of 40% and 38% in patients under 21 years of age after ACL reconstruction, compared to 9% and 4.2% in patients over 21 years of age in the same studies [29,30]. The rationale for the difference in rerupture rate in children compared to adults is not completely understood and likely multifactorial. One reason is certainly due to a higher activity index, as well as a higher return to sport rate of up to 87–96% [25,31] in children, compared to a rate of 65–75% [29] in adults. Furthermore, children might not adhere to sport restrictions and physiotherapy regimes as well as adults. Another reason might be more laxity of ligaments and joints in children.

In our cohort, patients without a rerupture up to the mid-term follow up had good clinical outcomes without significant difference in range of motion, strength, and functional tests compared to the contralateral knee. Patient-reported outcome measures, evaluated with the Lysholm, IKDC, and pedsQL scores, were good and similar to children treated with ACL reconstruction evaluated in a similar timeframe. In a systematic review by Longo et al. [11], including 53 studies using transphyseal and physeal sparing techniques for ACL reconstruction, the IKDC score was 91.68 (evaluated in 10 studies) versus 91.17 in our group, and Lysholm score was 96.25 (evaluated in 27 studies) versus 88.27 in our group. Unfortunately, we found no study introducing PedsQL after ACL reconstruction to compare our results to. The patient-reported outcomes in our study after 42 months are also comparable to those found in the adult population. Senftl et al. reported a median IKDC score of 89.4, and a median Lysholm score of 95 at 12 months postoperatively [23] in patients treated with DIS repair. In a study from Glasbrenner et al., comparing patient-reported outcomes in adults, they also did not find a difference between people treated with ACL repair and ACL reconstruction [32].

Long-term results of the patients with subjective intact ACL after 11 years showed good results, as well. Two patients (25%) reported a feeling of laxity, and two patients (25%) reported a reduced activity index. In the study by Glasbrenner et al., recurrent instability was reported in 35% of adult patients 5 years after ACL repair [32].

No radiographic signs of growth arrest were observed at 6 months. Long-term clinical follow up in the nine remaining patients revealed no patient-reported leg length discrepancies or axis deviations requiring treatment. Although there was no long-term radiological follow up, this might indicate that the implantation of the DIS device does not lead to relevant growth disturbances needing treatment.

Experimental data suggest that graft material and tunnel location influence physeal healing. Seil et al. [33] summarized that central growth plate lesion may result in a symmetric shortening, and the critical size for that was determined to be 7–9% of the size of the growth plate. No direct evidence exists regarding metallic transphyseal implants; in our cohort, centrally placed 10 mm Monobloc fixation was not associated with clinically relevant growth disturbance. One patient developed a genu valgum requiring varisation osteotomy at the time of revision surgery. Although a causal relationship to the Monobloc implantation cannot be excluded, experimental data suggest that angular deformities are more commonly associated with eccentric physeal injury, particularly at the femoral side, rather than centrally placed tibial lesions [33]. In the DIS technique, femoral drilling is performed in a manner comparable to standard transphyseal ACL reconstruction.

A macroscopic intact ACL during arthroscopy at hardware removal, and normal strength and stability in the follow up of patients who would later suffer a rerupture, show that the ACL healed at least to some degree. The high incidence of failure may suggest that DIS repair might just reflect the results of conservative treatment of ACL. However, in a meta-analysis by Ramski et al. [4], 75% of the children treated conservatively suffered from instability and laxity, and patient-reported outcomes and return to sport were significantly lower than in the children treated with the DIS technique.

This investigation is limited by its retrospective single-center design, the absence of a control group, and the small sample size. Three patients were treated after the suggested 21-day period for DIS repair; however, it did not lead to a higher reintervention rate in these three patients. Long-term follow ups were only patient-reported outcomes, and there was no long-term clinical testing or imaging performed; therefore, mild discrepancies were potentially missed. Comparisons were based on data from previously published reconstruction cohorts, which may differ in patient characteristics and follow-up duration.

Nevertheless, this study represents the first report to describe long-term outcomes of DIS repair in a pediatric and adolescent population.

## 5. Conclusions

DIS repair might support ACL healing with acceptable patient-reported mid- and long-term results. However, given the high failure rate of 55% and the high reintervention burden in our cohort, as well as the need to implant a relatively bulky Monobloc through an open physis, we currently do not recommend routine use of DIS repair in the pediatric population. Future prospective, multicenter studies with control groups are warranted to determine the true efficacy and long-term benefit of this technique in skeletally immature patients.

## Figures and Tables

**Table 1:** Patient demographics.

Patients	22
Female/male ratio	8 (36%)/14 (64%)
Age	13.26 years (range 9.33–15.83 years, median 13.92 years)
Time trauma-surgery	15.1 days (range 2–33 days, median 14 days)
Side	10 (45%) right, 12 (55%) left
Surgery/associated injury	9 (40%) only DIS
	7 (32%) additional repair lat. meniscus
	5 (23%) additional repair med. meniscus
	1 (5%) additional repair both menisci

**Table 2:** Patient details.

Patient	Age (years)	Sex	Side	Time Trauma to Surgery (days)	Type of Surgery	Revision	Reason for Revision	Time to Revision (years)	Type of Revision Surgery	Further Surgeries (Additional to Revision)
1 a,b	14.75	f	right	30	DIS, lat. menisc. repair	no				KA, HR
2 a,b	13.92	m	right	18	DIS, lat. menisc. repair	no				KA, HR
3	15.83	f	left	13	DIS, lat. menisc. repair	yes	rerupture (ice hockey)	1.92	ACL Reconstruction	1. KA, Debridement (before Revision) 2. KA, HR (before Revision)
4	14.75	m	left	14	DIS, med. menisc. repair	yes	rerupture (knee distorsion)	1	ACL Reconstruction, HR, med. menisc. partial Resection	-
5 a,b	13.83	m	right	25	DIS	no				HR
6 a	14.08	m	left	9	DIS	no				HR
7 a,b	14.83	m	left	14	DIS	no				-
8	14.08	f	left	8	DIS	yes	rerupture (football)	0.92	ACL Reconstruction, HR, lat. menisc. Repair	-
9	13.08	m	right	2	DIS, med. menisc. repair	yes	rerupture (wrestling)	1.08	ACL Reconstruction, HR, med. menisc. partial Resection	KA, medial menisc. Repair (after Revision)
10 a	13.5	m	right	4	DIS, lat. menisc. repair	yes	instability	5.25	ACL Reconstruction	KA, HR, lat. menisc. Repair (before Revision)
11 a	11.67	m	right	21	DIS, lat. menisc. repair	yes	rerupture (running)	7	ACL Reconstruction	KA, HR, lat. menisc. Repair (before Revision)
12	11.5	m	right	14	DIS	yes	rerupture (bicycle crash)	2.67	ACL Reconstruction	KA, HR (before Revision)
13 a	13.92	m	left	16	DIS	yes	instability	4	ACL Reconstruction	KA, HR (before Revision)
14 a,b	10	f	left	21	DIS	no				KA, HR
15	14.5	m	left	21	DIS, both menisc. repair	yes	rerupture (kneedistorsion)	3.25	ACL Reconstruction, med. menisc. Repair	1. KA, HR (before Revision) 2. KA, both menisc. repair (before Revision)
16 a,b	13.92	m	right	9	DIS, lat. menisc. repair	no				-
17 a,b	14.75	f	left	12	DIS, med. menisc. repair	no				KA, HR and ACL Reconstruction other Knee
18	10.25	m	left	33	DIS	yes	rerupture (skiing)	1.92	ACL Reconstruction	KA, HR (before Revision)
19	9.33	f	left	11	DIS	yes	rerupture (minor trauma)	10.3	ACL Reconstruction, varization Osteotomy, OATS	KA, HR (before Revision)
20	10	m	right	11	DIS, med. menisc. repair	yes	rerupture (trampoline)	1.92	ACL Reconstruction, HR	-
21 b	13.5	f	right	15	DIS, lat. menisc. repair	no				-
22 b	15.75	f	left	11	DIS, med. menisc. repair	no				-

Abbreviations: DIS: Dinamic intraligamemtary Stabilization, lat: lateral, med: medial, menisc. Repair: meniscal Repair, KA: Knee Arthroscopy, HR: Hardware Removal, OATS: Ostechondral Autograft Transfer. a: did take part in follow up with back to sports tests after 42 months, b: did take part in long-term follow up after 11 years.

**Table 3:** Range of motion, strength, and functional tests.

Range of Motion and Strength		Functional Tests		
Flexion (11/14)		Single Hop Test(11/14)		
contralateral knee:	mean 145.5° (range 145–150°, median 145°)	contralateral knee:	mean 148.5 cm	(range 61–178 cm, median 153 cm)
operated knee:	mean 145.5° (range 145–150°, median 145°)	operated knee:	mean 144.4 cm	(range 59–188 cm, median 156 cm)
difference:	mean 0° (range 0°, median 0°)	difference:	mean −4.1 cm	(range −25–+13 cm, median −2 cm)
		LSI:	mean 96.8%	(range 80.5–108.5%, median 96.7%)
Neutral (11/14)		6m Hop Test (11/14)		
contralateral knee:	mean 0° (range 0°, median 0°)	contralateral knee:	mean 2.4 s	(range 1.9–3.6 s, median 2.3 s)
operated knee:	mean 0° (range 0°, median 0°)	operated knee:	mean 2.4 s	(range 1.8–4.2 s, median 2.2 s)
difference:	mean 0° (range 0°, median 0°)	difference:	mean 0.01s	(range −0.4–+0.6 s, median 0 s)
		LSI:	mean 99.9%	(range 82.6–117.3%, median 100%)
Extension (11/14)		Triple Hop Test (11/14)		
contralateral knee:	mean 13.6° (range 5–25°, median 10°)	contralateral knee:	mean 504.1 cm	(range 255–605 cm, median 538 cm)
operated knee:	mean 15.5° (range 5–25°, median 10°)	operated knee:	mean 494.4 cm	(range 270–622 cm, median 520 cm)
difference:	mean 1.8° (range 10°–−5°, median 0°)	difference:	mean −9.7cm	(range −82–+61 cm, median −5 cm)
		LSI:	mean 98.4%	(range 85.9–105.9%, median 99.1%)
Lachmann (11/14)		Crossover Hop Test (11/14)		
contralateral knee:	mean 3.2 mm (range 0.5–6 mm, median 3 mm)	contralateral knee:	mean 455 cm	(range 221–562 cm, median 457 cm)
operated knee:	mean 4.3 mm (range 0.5–7 mm, median 4 mm)	operated knee:	mean 460.2 cm	(range 260–572 cm, median 494 cm)
difference:	mean 1.1 mm (range 0–2.5 mm, median 1 mm)	difference:	mean 5.3 cm	(range −56–+100 cm, median 0 cm)
		LSI:	mean 102.4%	(range 88.2–122.2%, median 100%)
Strength (10/14)				
LSI:	mean 119.7% (range 94.1–192.8%, median 106.1%)			

**Table 4:** Long-term follow up.

Patient	Time to Follow Up (years)	Revision	Further Surgery	Capability of Physical Activity (Normal/Reduced)	Pain (Often, Sometimes, Seldom, Never)	Feeling of Instability	Leg Length Difference	Satisfaction
1	12.83	no	no	reduced	sometimes	no	no	content
2	12.5	no	no	normal	never	no	no	content
5	11.83	no	no	normal	never	no	no	content
7	10.75	no	no	normal	never	no	no	content
14	11.9	no	no	reduced	often	yes	no	not content
16	11.33	no	no	normal	sometimes	no	no	content
17	6.67	no	no	normal	never	no	no	content
21	10.83	no	no	normal	never	no	no	content
22	10.83	no	no	normal	seldom	yes	no	content

## Data Availability

The original contributions presented in the study are included in the article, further inquiries can be directed to the corresponding authors.
